# Racial Differences in the Prevalence of Autism Spectrum Disorder: A Systematic Review

**DOI:** 10.1007/s10803-024-06403-5

**Published:** 2024-06-28

**Authors:** Zachary Gallin, Ana M. Kolevzon, Abraham Reichenberg, Sidney H. Hankerson, Alexander Kolevzon

**Affiliations:** 1 Icahn School of Medicine at Mount Sinai, New York, NY USA; 2 Pitzer College, Claremont, CA USA; 3 Seaver Autism Center for Research and Treatment, Icahn School of Medicine at Mount Sinai, New York, NY USA; 4 Department of Psychiatry, Icahn School of Medicine at Mount Sinai, New York, NY USA; 5 Department of Environmental Medicine and Public Health, Icahn School of Medicine at Mount Sinai, New York, NY USA; 6 Department of Population Health Sciences & Policy, Icahn School of Medicine at Mount Sinai, New York, NY USA; 7 Department of Pediatrics, Icahn School of Medicine at Mount Sinai, New York, NY USA

**Keywords:** Autism spectrum disorder, Pervasive developmental disorder, Prevalence, Epidemiology, Racial disparities, Ethnicity

## Abstract

**Purpose:**

Racial differences in prevalence rates of autism spectrum disorder (ASD) have shifted in the United States (US) since the 1990s. This review addresses the nature and context of this shift and discusses potential contributing factors and areas for future research.

**Methods:**

Seventeen population-based epidemiological birth cohort studies on ASD prevalence in the US that included race as a variable are included in the review. Studies were identified via a keyword search on PubMed. To be included, studies were required to include race or ethnicity as a variable in the prevalence estimates, include at least 1000 cases with autism, and be published in English by June 3rd, 2023.

**Results:**

Results suggest that in nearly all birth cohorts prior to 2010, ASD prevalence rates were highest among White children. ASD prevalence rates among Black, Hispanic, and Asian/Pacific Islander (API) children (22.3, 22.5, and 22.2 per 1000, respectively) surpassed prevalence rates among White children (21.2 per 1000) in the 2010 birth cohort and continued to increase in the 2012 birth cohorts.

**Conclusions:**

There are persistent racial differences in ASD prevalence in the US, and these differences were inverted after 2010, when ASD prevalence among Black, Hispanic, & API children surpassed ASD prevalence among White children. Possible drivers of this racial repatterning of ASD prevalence include changes in ASD screening and diagnosis, changes to health insurance policy, changes to immigration policy, and increased education attainment by minority groups.

## Introduction

Autism spectrum disorder (ASD) is a group of childhood-onset neurodevelopmental disorders characterized by impairments in communication and social interaction and by restricted interests and repetitive behaviors (American Psychiatric Association, 2013). ASD prevalence has increased globally since the 1990s (Elsabbagh et al., 2012; Zeidan et al., 2022); in the United States (US), ASD prevalence increased from 1 in 150 children born in 1992 (Rice, 2007) to 1 in 36 children born in 2012 (Maenner et al., 2023). Increased screening for ASD, broadening of diagnostic criteria, increased access to diagnostic and treatment services, and increased public awareness have significantly contributed to the increase in prevalence (Hansen et al., 2015; Maenner et al., 2023; Wazana et al., 2007; Zeidan et al., 2022).

As the overall ASD prevalence has increased and the sociocultural landscape in the US has changed, racial differences in prevalence have also shifted: ASD was most prevalent among White children in the 1990s but is now most prevalent among Black, Hispanic, and Asian/Pacific Islander (API) children (Maenner et al., 2023; Nevison & Parker, 2020). While recent review papers focus mainly on understanding and addressing the structural and sociocultural inequities that drive racial disparities in diagnosis, treatment, and service utilization (Aylward et al., 2021; Brasher et al., 2022; Pham & Charles, 2023), no study has yet to systematically examine when, how, and why the shift in ASD prevalence by race group occurred.

Autism was initially described by Leo Kanner in 1943 (Kanner, 1943) and then first appeared in the *Diagnostic and Statistical Manual of Mental Disorders, 3rd edition* (DSM-III) as ‘autistic disorder,’ defined as lack of responsiveness to other people, gross language deficits, peculiar speech, and bizarre behaviors with an onset before 30 months (American Psychiatric Association, 1980). Initial prevalence estimates were reported to be approximately 4.5 in 10,000 (Lotter, 1966). Since that time, prevalence rates have steadily risen, and several factors are thought to contribute to this increase.

One important factor is changing diagnostic criteria. With the release of the DSM-III-R in 1987, the definition of autistic disorder broadened to encompass early childhood-onset symptoms characterized by impaired social interaction, communication, and imaginative activity, and by restricted interests and activities (American Psychiatric Association, 1987). Even this subtle shift in language resulted in a likely increased rate of autism diagnosis (Volkmar et al., 1988). In 1994, with the publication of the DSM-IV, autistic disorder became encompassed within a larger category called pervasive developmental disorders (PDD), which also included Asperger’s syndrome, childhood disintegrative disorder, PDD Not Otherwise Specified (PDD-NOS), and Rett syndrome (American Psychiatric Association, 1994). PDD-NOS was a subthreshold diagnosis applied when autism-related features were present but full criteria for autistic disorder or Asperger’s syndrome were not met. Indeed, population-based epidemiological studies at the time showed that among the PDDs, PDD-NOS was the most prevalent (Chakrabarti & Fombonne, 2005). With the release of the DSM-5 (American Psychiatric Association, 2013), all the PDDs except for Rett syndrome were combined under a new umbrella term, autism spectrum disorder (ASD).

Other factors contributing to increasing prevalence rates are enhanced screening and younger age of diagnosis. The diagnosis of ASD can usually be established reliably by 24 months (Lord et al., 2006), and to receive an ASD diagnosis, children are evaluated by a multidisciplinary team that typically includes a child psychologist, developmental pediatrician, child psychiatrist, or another professional with expertise in diagnosing ASD (Zwaigenbaum et al., 2015). The gold standard for the evaluation of ASD includes a developmental history, a direct observational assessment of ASD symptoms, and application of DSM-5 criteria. Standardized assessments of adaptive functioning, language, and cognitive abilities are also typically performed (Sanchack & Thomas, 2016). Beginning in 2007, the American Academy of Pediatrics (AAP) recommended that pediatricians screen toddlers for ASD at age 18 months and 24 months using a validated and specific tool (Johnson et al., 2007). This recommendation led to increased early detection; children with ASD who screen positive on the Modified Checklist for Autism in Toddlers with Follow-Up (M-CHAT/F) are diagnosed 7.45 months earlier on average than children who screen negative (Guthrie et al., 2019). Earlier age of diagnosis increases ASD prevalence rates in population-based birth cohort studies (Hertz-Picciotto & Delwiche, 2009).

Regulatory factors also have a significant impact on ASD prevalence. The Individuals with Disabilities Education Act (IDEA) defines eligibility criteria for receiving special education services in the US. Prior to 1990, autism did not necessarily entitle one to specialized education, whereas intellectual disability (ID) did. IDEA was revised in 1991 to include ASD as a serviceable diagnosis (Education of the Handicapped Act Amendments of, 1990), and subsequently diagnostic substitution led to increased rates of the ASD diagnosis with decreasing rates of ID. For example, one population-based birth cohort study in California found the prevalence of autistic disorder to increase from 5.8 to 14.9 per 10,000 between 1991 and 1994 while the prevalence of ID decreased correspondingly from 28.8 to 19.5 per 10,000 over the same time period (Croen, Grether, Hoogstrate et al., 2002).

In addition to increases in overall rates of ASD diagnosis, prevalence varies between socioeconomic and cultural groups. For example, studies have shown that ASD prevalence in the US varies by primary spoken language (Dickerson, 2018), insurance status (King & Bearman, 2011; Winter et al., 2020), parental educational attainment (Croen et al., 2002b; Dickerson et al., 2017; King & Bearman, 2011; Winter et al., 2020), and neighborhood wealth (Dickerson et al., 2017; Durkin et al., 2017; King & Bearman, 2011; Mazumdar et al., 2013; Nevison & Parker, 2020). ASD prevalence in the US also varies by Black, White, API, and American Indian/Native American (AI/NA) race and by Hispanic versus non-Hispanic ethnicity. It is thought that these variations are driven by racial disparities in access to ASD diagnostic and healthcare resources (Aylward et al., 2021; Pham & Charles, 2023).

ASD is primarily considered genetic in origin with heritability estimates ranging from 70 to 90% (Bailey et al., 1995; Steffenburg et al., 1989). The genetic landscape is comprised of both common variants and rare de novo and inherited variants, with potentially hundreds of loci contributing to the phenotype through a polygenic inheritance pattern (Ramaswami & Geschwind, 2018). Gene discovery efforts have failed to include substantial numbers of non-White individuals, so it is unclear whether genetic drivers of ASD prevalence vary by race or ethnicity (Hilton et al., 2009). However, worldwide ASD prevalence does not vary significantly based on geographic region or ethnicity, so genetic drivers of ASD prevalence are likely constant between racial and ethnic groups (Elsabbagh et al., 2012).

Racial health disparities occur when health outcomes and access to necessary healthcare services differ between racial and ethnic groups (National Institute on Minority Health and Health Disparities, 2022). Racial health disparities are common in medicine (Bayne et al., 2023; Lewsey & Breathett, 2021; Owen et al., 2013; Richardson et al., 2003), including in the field of psychiatry (Akinhanmi et al., 2018; Garb, 2021; Garrett et al., 2023). For instance, Black and Hispanic Americans receive mental health care less often than White Americans, and disparities in access to mental health care between Black, Hispanic, and White Americans increased in the US between 2004 and 2012 (Cook et al., 2017). Moreover, Black patients are more than twice as likely to be diagnosed with schizophrenia compared to White patients (Olbert et al., 2018) but are less likely to receive clozapine, the only FDA-approved drug for treatment-resistant schizophrenia (Ventura et al., 2022).

Racial health disparities permeate ASD screening, diagnosis, and treatment. Discrimination by healthcare providers, cultural stigma, income disparities, differences in citizenship status, and a shortage of multilingual clinicians present obstacles for racial and ethnic minority groups seeking ASD assessment and treatment (Brasher et al., 2022). Accordingly, Black and Hispanic children have historically been diagnosed with ASD at an older age than White children (Mandell et al., 2002; Valicenti-McDermott et al., 2012), although recent research suggests that this disparity is closing (Maenner et al., 2020). Similarly, among a cohort of 406 Medicaid-eligible children in Philadelphia, Black children with ASD were 2.6 times more likely to be misdiagnosed; they were 5.1 times more likely to be diagnosed with adjustment disorder and 2.4 times more likely to be diagnosed with conduct disorder prior to ASD diagnosis (Mandell et al., 2007). In another study using semi-structured interviews of female caregivers of Black children with ASD, “the majority of caregivers felt racism affected their experiences seeking primary healthcare services for their child with ASD” (Dababnah et al., 2018).

Cultural stigma and lack of awareness surrounding ASD may also impede diagnosis among Black and Hispanic children. At least three different qualitative studies have reported that Black and Hispanic/Latino parents of children with ASD face stigma and social isolation, which can dissuade them from accepting referrals for ASD symptoms (Cohen & Miguel, 2018; Dababnah et al., 2018; Ijalba, 2016). Other qualitative studies document that Hispanic/Latino parents have limited awareness of ASD and may assume that signs that indicate elevated risk for ASD are instead characteristic of typical development (Ijalba, 2016; Zuckerman et al., 2014).

The aim of this review is to summarize temporal trends in ASD prevalence by race group among children born in the US between 1987 and 2016 by evaluating population-based epidemiological studies, and to clarify the implications of this shift. These results will complement existing knowledge on racial disparities in ASD diagnosis and provide a foundation for future research on associated factors.

## Methods

A systematic review of autism prevalence by race in the US was conducted according to the PRISMA criteria (Page et al., 2021). A keyword search was done on PubMed using the following search terms: ‘autism’ or ‘ASD’ and ‘prevalence’ and ‘race’ or ‘ethnicity’ in the title, abstract, and keywords sections. Additional studies were identified in the reference sections of review papers found in the keyword search. To be included in this review, studies were required to (1) be population-based epidemiological birth cohort studies conducted in the US, (2) include race or ethnicity as a variable in the prevalence estimates, (3) include at least 1000 cases with autism, (4) be published in English by June 3rd, 2023. Three reviewers independently screened each study for inclusion in the review, assessed each study for potential bias, and extracted study data. Risk of bias in each study was assessed using the ROBINS-I tool (Sterne et al., 2023). The review was not registered, and a protocol was not prepared.

## Results

**Fig. 1:**
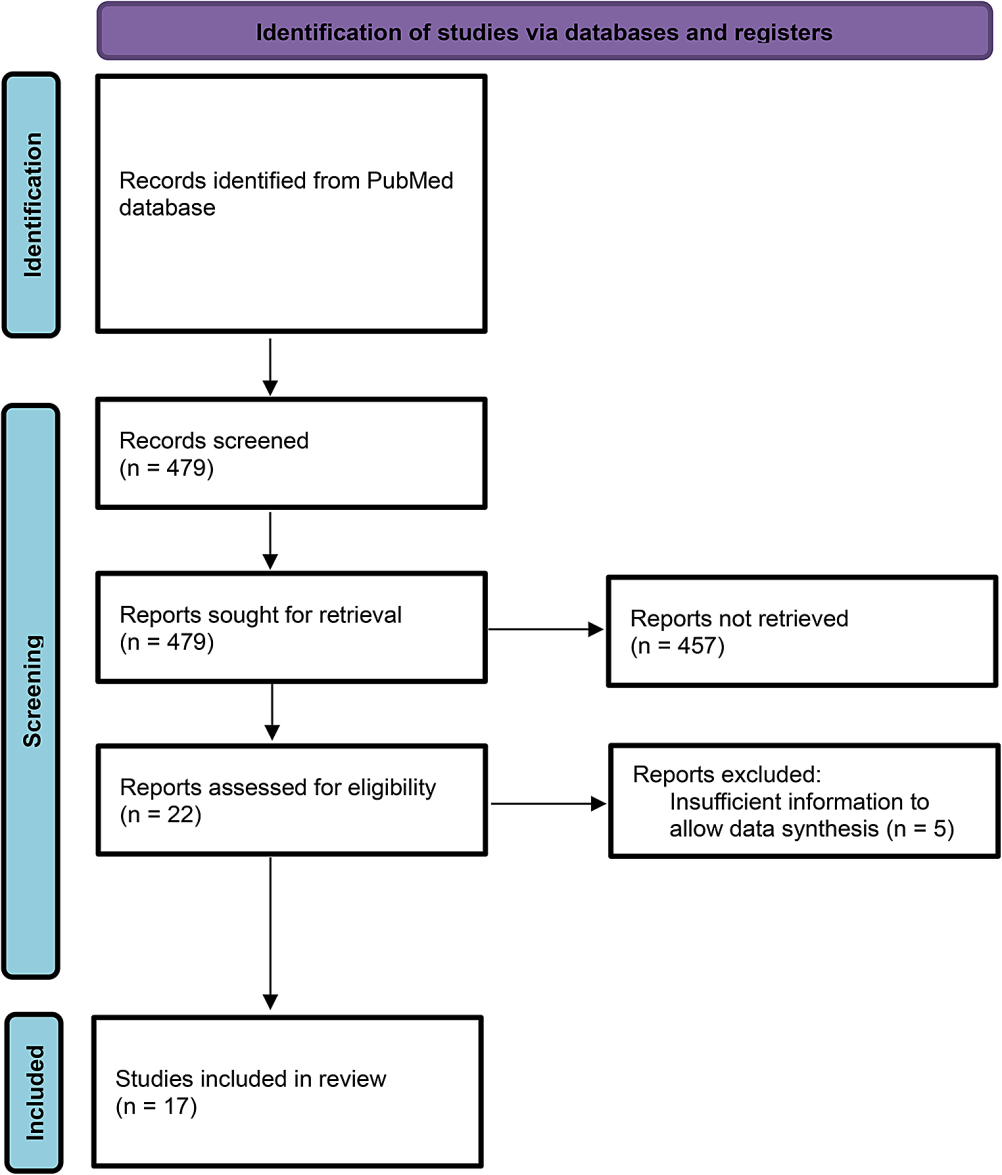
PRISMA 2020 flow diagram for new systematic reviews which included searches of databases and registers only. *Adapted from* Page et al., 2021

479 articles were identified using the keyword search criteria. Of these, twenty-two studies were identified that met our *a priori* criteria. Five of the twenty-two studies (Braun et al., 2015; Christensen et al., 2019; Dickerson et al., 2017; Jarquin et al., 2011; Pedersen et al., 2012) were excluded because of insufficient information to allow data synthesis. Seventeen studies remained and are included in this review (Fig. 1). Data on ASD prevalence by race group in a set year or series of years was retrieved from each study. Prevalence values were converted to cases per 1000 population when necessary. In one of the included studies (Rice, 2007), data stratified by state were combined during the review process to attain a sample size greater than 1000 cases with ASD. All studies included in the review were determined to have a moderate risk of bias due to confounding by insurance coverage, education level, and socioeconomic status. Study characteristics, results, and confidence intervals for each included study are presented in Tables 1 and 2.

**Table 1:** ADDM Articles on ASD Prevalence by Race Group 1994–2015

Author & Date	Sample Size	Birth Year	Sites Included	Prevalence per 1000 or Odds Ratio	Conclusions
Rice, 2007	1,148	1992	AZ, GA, MD, NJ, SC	Non-Hispanic White: 8.0 (7.4–8.6) Non-Hispanic Black: 6.5 (5.8–7.2)	ASD prevalence is higher in White children than in Black children.
Mandell et al., 2009^1^	1,497	1994	AL, AZ, AR, CO, GA, MD, MO, NJ, NC, PA, SC, UT, WI, & WV	Non-Hispanic White: control Non-Hispanic Black: 0.79 (0.64–0.96) Hispanic: 0.76 (0.56–0.99)	White children are more likely to be diagnosed with ASD than Black & Hispanic children.
Durkin et al., 2017^1^	2,338	1994	AL, AZ, AR, CO, GA, MD, MO, NJ, NC, UT, & WI	Non-Hispanic White: 6.7 (6.4-7.0) Non-Hispanic Black: 5.9 (5.4–6.5) Hispanic: 3.9 (3.4–4.5)	ASD prevalence is higher in Black & White children than in Hispanic children.
Rice, 2009	2,757	1998	AL, AZ, CO, GA, MD, MO, NC, PA, SC, & WI	Non-Hispanic White: 9.9 (9.4–10.4) Non-Hispanic Black: 7.2 (6.6–7.8) Hispanic: 5.9 (5.3–6.6)	ASD prevalence is highest in White children, then Black children, then Hispanic children.
Baio, 2012	3,820	2000	AL, AZ, AR, CO, FL, GA, MD, MO, NJ, NC, PA, SC, UT, WI, & WV	Non-Hispanic White: 12.0 (11.5–12.5) Non-Hispanic Black: 10.2 (9.5–10.9) Hispanic: 7.9 (7.2–8.6)	ASD prevalence is highest in White children, then Black children, then Hispanic children.
Baio, 2014	5,338	2002	AL, AZ, AR, CO, GA, MD, MO, NJ, NC, UT, & WI	Non-Hispanic White: 15.8 (15.2–16.3) Non-Hispanic Black: 12.3 (11.5–13.1) Hispanic: 10.8 (10.0-11.6) API: 12.3 (10.7–14.2)	ASD prevalence is higher in White children than in Black, Hispanic, & API children.
Christensen et al., 2016	5,063	2004	AZ, AR, CO, GA, MD, MO, NJ, NC, SC, UT, & WI	Non-Hispanic White: 15.3 (14.7–15.8) Non-Hispanic Black: 13.1 (12.3–13.9) Hispanic: 10.2 (9.4–10.9) API: 11.4 (9.9–13.1)	ASD prevalence is highest in White children, then Black children, then Hispanic children. It is higher in White children than in API children.
Baio et al., 2018	5,473	2006	AZ, AR, CO, GA, MD, MN, MO, NJ, NC, TN, & WI	Non-Hispanic White: 17.2 (16.5–17.8) Non-Hispanic Black: 16.0 (15.1–16.9) Hispanic: 14.0 (13.1–14.9) API: 13.5 (11.8–15.4)	ASD prevalence is higher in Black & White children than in Hispanic children. It is higher in White children than in API children.
Maenner et al., 2020	3,981	2008	AZ, AR, CO, GA, MD, MN, MO, NJ, NC, TN, & WI	Non-Hispanic White: 18.5 (17.9–19.3) Non-Hispanic Black: 18.3 (17.2–19.4) Hispanic: 15.4 (14.4–16.4) API: 17.9 (15.9–20.1)	ASD prevalence is higher in Black & White children than in Hispanic children.
Maenner et al., 2021	5,058	2010	AZ, AR, CA, GA, MD, MN, MO, NJ, TN, UT, & WI	Non-Hispanic White: 21.2 (20.3–22.0) Non-Hispanic Black: 22.3 (21.0-23.7) Hispanic: 22.5 (21.2–23.9) API: 22.2 (19.8–24.8)	ASD prevalence is equal among White, Black, Hispanic, & API children.
Maenner et al., 2023	6,245	2012	AZ, AR, CA, GA, MD, MN, MO, NJ, TN, UT, & WI	Non-Hispanic White: 24.3 (23.4–25.2) Non-Hispanic Black: 29.3 (27.9–30.9) Hispanic: 31.6 (30.0–33.3) API: 33.4 (30.5–36.4) two or more races: 22.9 (20.3–25.8)	ASD prevalence is higher among API, Hispanic children, & Black children than among White children & children of two or more races.
Shaw et al., 2020	1,125 Age: 4	2012	AZ, CO, MO, NJ, NC, & WI	Non-Hispanic White: 13.2 (12.0-14.4) Non-Hispanic Black: 14.3 (12.5–16.3) Hispanic: 18.4 (16.5–20.5)	ASD prevalence is higher in Hispanic children than in Black & White children.
Shaw et al., 2023^2^	179,699 Age: 3–21	1997–2015	AZ, AR, MN, NJ, TN, UT, & WI	Non-Hispanic White: 17 (17–18) Non-Hispanic Black: 15 (15–15) Hispanic: 13 (13–13) API: 14 (14–14) AI/AN: 13 (12–13)	ASD prevalence is highest in White children, then Black children, then API children, then Hispanic & AI/AN children.

*Abbreviations* ASD = autism spectrum disorder; ADDM = Autism and Developmental Disabilities Monitoring Network; AL = Alabama; AZ = Arizona; AR = Arkansas; CA = California; CO = Colorado; FL = Florida; GA = Georgia; MD = Maryland; MN = Minnesota; MO = Missouri; NJ = New Jersey; NC = North Carolina; TN = Tennessee; PA = Pennsylvania; SC = South Carolina; UT = Utah; WI = Wisconsin; WV = West Virginia.

^1^Mandell et al. (2009) and Durkin et al. (2017) report on the same ADDM Network surveillance data from AZ, AR, CO, GA, MD, MO, NJ, NC, UT, & WI and therefore have similar results. Mandell et al. (2009) present the results as odds ratios, while Durkin et al. (2017) present the results as prevalence per 1000.

^2^The study population for Shaw et al. (2023) includes all children aged 3–21 living in AZ, AR, MN, NJ, TN, UT, or WI in 2018, so it overlaps with the study populations in every other ADDM study. As such, their study may be interpreted as a complement to the other ADDM studies that discusses overall ASD prevalence by race in certain states with ADDM sites.

**Table 2:** Non-ADDM Articles on ASD Prevalence by Race Group 1987–2016

Author & Date	Location & Sample Size	Participant Ages & Birth Years	Data Source	Diagnostic Criteria	Prevalence per 1000 or Odds Ratio	Conclusions
Sullivan, 2013	US (excluding AK, MA, NJ, VT, & DC), Sample size: 272,311	Age: 6–21 Birth year: 1987–2002	School record - IDEA S618	Variable	White: 4.8 Black: 4.1 Hispanic: 2.9 AI/AN: 3.6 API: 5.8	ASD prevalence is highest in API children, then White children, then Black children, then AI/AN children, then Hispanic children.
Croen et al., 2002^1^	California, Sample size: 4,381	Age: 0–10 Birth year: 1989–1994	Medical record – California DDS	DSM-IIIR	White: control Hispanic: 0.6 (0.5–0.6) Black: 1.2 (1.1–1.3) Asian: 1.1 (1.0-1.3)	Black children are more likely to be diagnosed with autistic disorder than White children, Asian children are equally likely, and Hispanic children are less likely.
Winter et al., 2020^1^	California, Sample size: 122,392	Age: 3–6 Birth year: 1992–2016	Medical record – California DDS	DSM-5	See table S4 in appendix for yearly prevalence by race	Autism prevalence rates have plateaued in White communities but continue to increase in communities of color.
Pham et al., 2022^2^	US (excluding RI), Sample size: 48,501	Age: 8 Birth year: 2009–2013	Medical record - Cosmos	ICD-10-CM	See table in article for yearly prevalence by race	ASD prevalence was highest among White children born in 2009 but highest among Black children born in 2013, with a gradual flip between 2009 and 2013.

*Abbreviations* ASD = autism spectrum disorder; ADDM = Autism and Developmental Disabilities Monitoring Network; US = United States of America; AK = Alaska, MA = Massachusetts; NJ = New Jersey; VT = Vermont; DC = District of Columbia; RI = Rhode Island; ICD = International Classification of Diseases; DSM = Diagnostic and Statistical Manual of Mental Disorders; IDEA = Individuals with Disabilities Education Act.

^1^The study population in Croen et al. (2002) paper includes all Californians aged 0–10 in 1994, so it overlaps with children born in 1992–1994 included in Winter et al.’s (2020) paper. However, this has little impact on study inference, as most children likely were not diagnosed with ASD by age 2.

^2^ The study population for Pham et al. (2022) includes children born between 2009 and 2013 in every US state except for RI, so it may overlap with the study populations for Winter et al. (2020), Shaw et al. (2020, 2023), and Maenner et al. (2021, 2023). The potential for data overlap must be considered when examining Pham et al.’s results alongside the other studies.

**Fig. 2:**
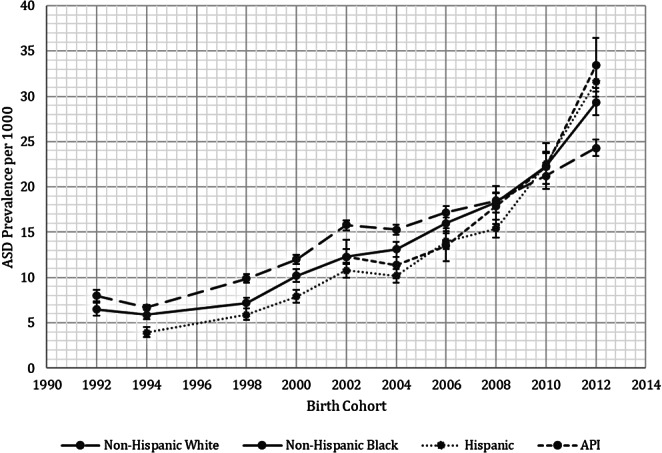
ADDM Data on Time Trends in ASD Prevalence by Race Group 1992–2012. *Error bars represent 95% confidence intervals (CIs). All data and CIs were originally reported in the ADDM reports except for the 1992 birth cohort, in which prevalence was calculated as a proportion using data from Rice (2007), with CIs derived from the binomial calculation

Thirteen of the seventeen studies are affiliated with the Centers for Disease Control (CDC) Autism and Developmental Disabilities Monitoring (ADDM) Network (Baio, 2012, 2014; Baio et al., 2018; Christensen et al., 2016; Durkin et al., 2017; Maenner et al., 2020, 2021, 2023; Mandell et al., 2009; Rice, 2007, 2009; Shaw et al., 2020, 2023). ADDM has reported ASD prevalence in sites located across the US every other year since 2000. Each site monitors ASD prevalence among 8-year-olds in a specific geographic area of its state. Some ADDM sites have also monitored ASD prevalence among 4-year-olds starting in surveillance year 2014 (Christensen et al., 2019; Shaw et al., 2020). Sites ascertain ASD prevalence using medical and/or public school records. Any child with a documented diagnosis of ASD (or PDD prior to 2013), enrollment in special education services with a classification of ASD, or an International Classification of Diseases code consistent with ASD (e.g., F84 or 299) is considered a potential ASD case. In surveillance years 2000–2016, ADDM Network clinicians conducted deidentified chart review on all potential ASD cases to confirm that each case met the DSM criteria for ASD before adding it to the ASD prevalence counts. Since 2018, all potential ASD cases have been included in the ASD prevalence counts. Data on race and ethnicity of cases are derived from medical records, school records, birth certificate linkages, or administrative and billing information. Population denominators are obtained from the National Center for Health Statistics race estimates or from the most recent US census and include categories for AI/AN, API, Black, White, and two or more races, and Hispanic ethnicity. Any case with Hispanic ethnicity is excluded from all other racial groups. Results from the thirteen included ADDM studies are collectively represented in a graph of ASD prevalence by race group over time (Fig. 2).

Across race groups, ASD prevalence is higher in males (43.0 per 1000) than in females (11.4 per 1000) (Maenner et al., 2023). According to data collected by the ADDM Network, ASD prevalence has increased from 6.2 per 1000 among children born in 1994 (Durkin et al., 2017) to 27.6 per 1000 among children born in 2012 (Maenner et al., 2023). The ten biannual ADDM reports included in the review show that ASD prevalence was originally highest among White children, but this pattern inverted for children born in 2008 or later. Reports on birth cohorts before 2008 indicate that ASD prevalence among White children was higher than ASD prevalence among children from any other racial group, while prevalence among Hispanic children was lower than ASD prevalence among children from any other group (Baio, 2012, 2014; Baio et al., 2018; Christensen et al., 2016; Durkin et al., 2017; Maenner et al., 2020; Mandell et al., 2009; Rice, 2007, 2009). For instance, in the 1994 birth cohort, ASD prevalence was 6.7 (95% confidence interval (CI) 6.4-7.0) per 1000 among White children, while it was 5.9 (CI 5.4–6.5) and 3.9 (CI 3.4–4.5) per 1000 among Black and Hispanic children, respectively (Durkin et al., 2017). Therefore, in the 1994 birth cohort, the Black to White odds ratio (OR) was 0.79 (CI 0.64–0.96), while the Hispanic to White OR was 0.76 (CI 0.56–0.99) (Mandell et al., 2009). In the 2010 birth cohort, ASD prevalence among White children was 21.2 (CI 20.3–22.0) per 1000. This was roughly equal to ASD prevalences among Black, Hispanic, and API children, which were 22.3 (CI 21.0-23.7), 22.5 (CI 21.2–23.9), and 22.2 (CI 19.8–24.8) per 1000, respectively, resulting in a White to Black prevalence ratio (PR) of 0.9 (CI 0.9-1.0), a White to Hispanic PR of 0.9 (CI 0.9-1.0), and a White to API PR of 1.0 (CI 0.8–1.1) (Maenner et al., 2021). The most recent ADDM report from the 2012 birth cohort reveals that the racial patterning of ASD prevalence in the US has fundamentally shifted; ASD prevalence among White children is now 24.3 (CI 23.4–25.2) per 1000, lower than ASD prevalences among Black, Hispanic, and API children, which are 29.3 (CI 27.9–30.9), 31.6 (CI 30.0–33.3), and 33.4 (CI 30.5–36.4) per 1000, respectively. Accordingly, the Black to White PR is now 1.2 (CI 1.1–1.3), while the Hispanic to White PR is 1.3 (CI 1.2–1.4) and the API to White PR is 1.4 (CI 1.2–1.5) (Maenner et al., 2023) (Table 1; Fig. 2).

Two of the eighteen studies included in the review are analyses of medical records from the California Department of Developmental Services (DDS) (Croen et al., 2002b; Winter et al., 2020). Individuals with ASD in California can enroll in the DDS to receive support services, and most Californians with ASD are enrolled. ASD diagnoses are confirmed by healthcare professionals at DDS centers in California. Before the release of the DSM-5, the DDS included individuals with autistic disorder but not Asperger’s syndrome or PDD-NOS in its autism case counts. In 2014, the DDS began to include all individuals who met the DSM-5 definition of ASD in its case counts.

The California DDS studies support the ADDM Network’s findings. Croen et al. (2002) examine ASD prevalence among 0-10-year-olds from a 1989–1994 birth cohort and find that Hispanic children were less likely to be diagnosed with autistic disorder than White and Asian children, with an OR of 0.6 (CI 0.5–0.6), while Black children were more likely to be diagnosed, with an OR of 1.2 (CI 1.1–1.3). While this finding among Black children is not consistent with the early ADDM studies, the results among Hispanic children are consistent. Winter and colleagues (2020) find that autism prevalence among 3-6-year-olds was lower in children of Hispanic mothers than in children of White mothers born from 1992 to 2008 but has been lowest in children of White mothers born after 2011 due to increased prevalence among children of Hispanic, Black, and Asian mothers. For instance, in the 1992 birth cohort, ASD prevalence was 4.5 (CI 4.2–4.8) per 1000 among children of White mothers, while it was 2.2 (CI 2.0-2.4) per 1000 among children of Hispanic mothers. In the 2012 birth cohort, ASD prevalence was 13.0 (CI 12.4–13.6) per 1000 among children of White mothers, while it was 14.5 (CI 14.0–15.0) per 1000 among children of Hispanic mothers (Winter et al., 2020). This finding may be confounded by the broadening of the California DDS diagnostic criteria from autistic disorder to ASD in 2014, and Winter et al.’s (2020) study differs from other studies covered by this review in that it categorizes children by maternal race, resulting in potential differences in the categorization of biracial children. The studies nevertheless support the ADDM Network findings that ASD prevalence in the Hispanic and API race groups surpassed ASD prevalence in the White race group around 2010 (Table 2).

Shifts in ASD/PDD prevalence by race around 2010 are also reflected in nationwide non-ADDM analyses of school and medical records. Analysis of IDEA school records for 6-21-year-olds born in 1987–2002 indicates that prevalence of enrollment in special education services for ASD was highest in API children, then White children, then Black children, then AI/AN children, and lowest among Hispanic children (Sullivan, 2013). Analysis of medical records from Cosmos, a dataset defined by the Health Insurance Portability and Accountability Act, indicates that ASD prevalence among 8-year-olds was highest among White children born in 2009 but highest among Black children born in 2013, with a gradual shift in rates between 2009 and 2013 (Pham et al., 2022) (Table 2).

## Discussion

This review suggests that there are persistent racial differences in ASD prevalence in the US, and that these differences appear to have inverted in children born after 2010, when ASD prevalence among Black, Hispanic, & API children surpassed ASD prevalence among White children. Studies from the United Kingdom (UK), Israel, and Western Australia also identify differences in ASD prevalence between race groups in their respective countries, but they are difficult to compare to US-based studies because each country defines its race groups differently. Furthermore, none of these studies allow for comparison of time trends in ASD prevalence by race. A UK-based case-control prevalence cohort study suggests that ASD prevalence is lowest among Roma children, then Asian children, then Chinese children, then White children, and highest among Black children (Roman-Urrestarazu et al., 2021). A total population retrospective study (Raz et al., 2015) and total population nested case-control study (Segev et al., 2019) from Israel argue that ASD prevalence is lower among Arabs than the general population. A retrospective cohort study from Western Australia finds that ASD prevalence is lower among aboriginal children than Caucasian children (Fairthorne et al., 2017).

The shift in comparative rates of ASD prevalence by race group in the US may be due to improvements in ASD diagnosis in racial minorities. Data suggests that the gap in age at time of ASD diagnosis between Black, Hispanic, and White children in the US is closing. In a 1993–1999 birth cohort of 406 Medicaid-eligible children with ASD in Philadelphia, Pennsylvania, Black children were diagnosed 1.6 years later than White children on average (Mandell et al., 2002). In a 2003–2010 birth cohort of 399 children with ASD in The Bronx, New York, Black children were diagnosed seven months later than White children on average, while Hispanic children were diagnosed five months later than White children (Valicenti-McDermott et al., 2012). In the 2008 ADDM birth cohort, Black children were diagnosed just two months later than White children on average, while Hispanic children were diagnosed at the same age as White children (Maenner et al., 2020). These improvements may be driven by policy changes that made ASD diagnosis more accessible, such as the expansion of IDEA in 1991 and the AAP’s ASD screening guidelines in 2007. However, healthcare providers may also be over-diagnosing Black and Hispanic children with ASD. In a survey of 400 behavioral pediatricians with clinical interest in ASD, 58% of providers stated that their colleagues at least sometimes over-diagnose ASD (Azim et al., 2020). More research is needed to explore whether ASD over-diagnosis in racial minorities has contributed to the racial repatterning of ASD prevalence.

The passing of the Affordable Care Act (ACA) in 2010 led to changes in health insurance coverage in the US that may also have played a role in the racial repatterning of ASD prevalence. Rates of insurance coverage among all race groups substantially increased after implementation of the ACA, while racial disparities in insurance coverage decreased. Between 2010 and 2021, the uninsured shares of the nonelderly Black, Hispanic, API, & AI/AN populations fell by 9%, 14%, 11%, and 11%, respectively, while the uninsured share of the nonelderly White population fell by only 6% (Artiga et al., 2022). Changes in insurance coverage have correlated with changes in healthcare utilization and reported ASD prevalence. Durkin and Wolfe (2020) reported that the ASD prevalence rate temporarily plateaued during the 2008 recession, when many children lost their insurance coverage as parents lost their jobs. Conversely, as rates of insurance coverage increased among individuals with severe psychological distress between 2011 and 2016, fewer individuals reported delaying necessary care, forgoing care, and being unable to afford mental health care (Novak et al., 2018). As rates of insurance coverage increased in the general population between 2006 and 2014, a greater percent of Americans reported visiting a physician in the past year, although the percent of Americans who reported visiting a mental health provider in the past year stayed constant (Manuel, 2018).

Various other policies included in or related to the ACA may have increased the rate of ASD diagnosis, especially among marginalized populations. First, the ACA prevented insurers from denying coverage to patients with preexisting conditions, including ASD. Similarly, autism mandates have been enacted across all 50 US states between 2001 and 2020, requiring private insurers to expand coverage for ASD on a state-by-state basis (Choi et al., 2020). Policy changes also resulted in increased access to ASD-specific health services. Some states expanded access to home care services for children with ASD in the 2000s and 2010s using federal 1915(c) waivers (Velott et al., 2016). Then, the ACA mandated that private insurers cover the AAP-recommended ASD screenings at 18 months and 24 months of age without charging copays (US Department of Health and Human Services, n.d.). Furthermore, the ACA enabled many states to undergo Medicaid expansion, resulting in a significant increase in healthcare providers qualified to treat ASD in these states (McBain et al., 2022). Finally, the ACA helped address inequities in coverage for mental health services by strengthening the Mental Health Parity and Addiction Equity Act of 2008 (MHPAEA), which has been associated with improvements in access to ASD-specific services covered by insurance (Stuart et al., 2017).

Changes to immigration policy in the US likely also played a role in the racial repatterning of ASD prevalence. Fountain & Bearman (2011) argue that rates of ASD diagnosis among Hispanic children in California increased most rapidly when national policy was favorable to immigrants and rates of deportation of noncitizens from the US stayed constant. ASD diagnosis rates among Hispanics in California plateaued when policy changes like Proposition 187 drove increased deportations of noncitizens. Rates of apprehension, removal, return, and/or expulsion of noncitizens in the US steadily decreased between 2000 and 2015 but have been increasing since 2015 (US Department of Homeland Security, 2022). It is possible that the increased nationwide ASD prevalence among Hispanic children is related to decreasing rates of deportation of noncitizens in the 2000s and the scientific community has yet to detect the impact of increasing deportations since 2015 on ASD diagnosis rates and racial patterning.

Increased parental education has also been associated with a higher likelihood of ASD diagnosis (Croen et al., 2002b; Dickerson et al., 2017; King & Bearman, 2011). A few qualitative studies suggest that cultural factors such as stigma and lack of awareness may contribute to racial differences in ASD diagnosis (Cohen & Miguel, 2018; Dababnah et al., 2018; Ijalba, 2016; Zuckerman et al., 2014), but no US-based epidemiological studies have collected data on how cultural factors vary between race groups and how this has changed over time. As knowledge of racial differences in ASD prevalence has become widespread, various initiatives have cropped up to combat stigma and increase cultural awareness of ASD in minority communities (Dufour et al., 2021; Kamali et al., 2022; Kang-Yi et al., 2018). It is possible that cultural changes have contributed to the racial repatterning of ASD prevalence by encouraging more parents from minority race groups to follow up on ASD screening referrals. More research is needed to determine if the culture around ASD in minority race groups has changed and whether this is affecting reported ASD prevalence.

The patterning of ASD prevalence by neighborhood wealth has shifted throughout the US, and this shift may impact racial differences in ASD prevalence. In California, neighborhood wealth correlated with overall likelihood of ASD diagnosis before 1994 but only correlated with likelihood of ASD diagnosis among public insurance recipients from 1994 to 2000 (King & Bearman, 2011). County wealth was positively related to ASD prevalence among White children in California before 2000 but negatively related to ASD prevalence among White children after 2000 (Nevison & Parker, 2020). Similarly, parental college education and private insurance used to be associated with higher likelihood of ASD diagnosis in California, but these patterns flipped in 2009 and 2011, respectively. Californian children with public insurance and poorly educated parents are now more likely to be diagnosed with ASD than children with private insurance and well-educated parents (Winter et al., 2020). Likewise, ADDM data from the 1994 to 2002 birth cohorts suggests that ASD prevalence was higher in wealthy neighborhoods nationwide (Durkin et al., 2017), while ADDM data from the 2010 and 2012 birth cohorts concludes that neighborhood wealth had no effect on ASD prevalence (Maenner et al., 2021, 2023). Black and Hispanic Americans are more likely to live in low-income neighborhoods than White and Asian Americans (Reardon et al., 2015), and Black and Hispanic Californians are more likely to lack a college education (Hawkins, 2022) and private insurance (Finocchio et al., 2021). Therefore, the decreasing impact of neighborhood wealth on ASD prevalence along with the increasing rate of ASD diagnosis among Californians lacking a college education and private insurance may contribute to increasing ASD rates among Black and Hispanic children.

This review is limited by the heterogeneity of diagnostic criteria for autism across studies. Of the eighteen studies in this review, fourteen used either ASD or enrollment in special education services for their diagnostic criteria, one used only ASD, one used only enrollment in special education services, one used only autistic disorder, and one used autistic disorder before 2014 and ASD after 2014. ASD and PDD are equivalent diagnoses in the DSM-5 and DSM-IV, respectively, while autistic disorder is a narrower diagnosis in the DSM-IV that excludes most ASD cases. Criteria for enrollment in special education services under IDEA align closely, but not perfectly, with the DSM-5 definition of ASD. The USDOE defines autism as “a developmental disability significantly affecting verbal and nonverbal communication and social interaction, generally evident before age 3 that adversely affects a child’s educational performance” (US Department of Education, 2017). State governments can amend the USDOE definition of ASD to best serve their school districts. Therefore, enrollment in special education services for ASD isn’t always equivalent to ASD diagnosis (Sullivan, 2013).

In addition, this review is limited by discrepancies in how each study defines race groups. Two studies have a general White race group, while fifteen specify non-Hispanic White. Four studies have a general Black race group, while thirteen specify non-Hispanic Black. Fifteen studies have a general Hispanic race group, while one specifies Hispanic White and one lacks a Hispanic category. Two studies have a specific Asian race group, nine group together Asian and Pacific Islander (API) children, and six, including all ADDM reports before 2002, lack an Asian category. Only two studies include a group for AI/AN, and only one study includes a group for children of two or more races. Variations in sample size between race groups also affect the integrity of the data: in the 2008 and 2010 ADDM birth cohorts, the confidence interval for ASD prevalence among API children is so large that it overlaps with the confidence intervals for every other race group included (Maenner et al., 2020, 2021). Data from international epidemiological studies further suggest that race is socially constructed, so discussion of disease prevalence by race group reflects the structure and norms of the culture that conceptualized the race groups (Fairthorne et al., 2017; Raz et al., 2015; Roman-Urrestarazu et al., 2021; Segev et al., 2019).

The pattern of ASD prevalence in the US has fundamentally changed in recent years. ASD, which had been most prevalent among White children in the 1990s and early 2000s, is now more prevalent among Black, Hispanic, & API children. These results complement existing research on racial disparities in the diagnosis and treatment of ASD and raise important questions for future epidemiological studies on the relationship between ASD prevalence and race, ethnicity, social class, and socioeconomic status.

Many sociocultural and regulatory factors could have contributed to the rise in reported ASD prevalence among minority groups in the US. Younger age of diagnosis, increased education and wealth, and enhanced insurance coverage among Black and Hispanic families have significantly impacted the racial patterning of ASD prevalence in the US. At the same time, healthcare providers may be disproportionately over-diagnosing ASD in Black and Hispanic children in an effort to improve access to care. Despite the limitations in interpreting findings from studies with heterogeneous methods and outcome classifications, there is adequate consistency across studies to suggest a clear shift, where ASD prevalence among Black, Hispanic, & API children in the US born after 2010 surpassed that of White children.

Given that race is culturally defined and varies depending on social norms, the implications of this shift in prevalence are challenging to fully understand. Race often acts as a proxy for variables like genetics, access to healthcare resources, and exposure to racism. There is a need for future rigorous epidemiological studies on the genetic, sociocultural, and economic drivers of trends in ASD prevalence that include population samples representative of the racial diversity of the US population.
